# DNA and RNA Stability of Marine Microalgae in Cold-Stored Sediments and Its Implications in Metabarcoding Analyses

**DOI:** 10.3390/ijms25031724

**Published:** 2024-01-31

**Authors:** Zhaoyang Chai, Yuyang Liu, Siyang Jia, Fengting Li, Zhangxi Hu, Yunyan Deng, Caixia Yue, Ying-Zhong Tang

**Affiliations:** 1 CAS Key Laboratory of Marine Ecology and Environmental Sciences, Institute of Oceanology, Chinese Academy of Sciences, Qingdao 266071, China; zhaoyangchai@qdio.ac.cn (Z.C.); lyy9303130@163.com (Y.L.); lifengting111@126.com (F.L.); huzx@gdou.edu.cn (Z.H.); yunyandeng@qdio.ac.cn (Y.D.); yuecaixia@qdio.ac.cn (C.Y.); 2 Laoshan Laboratory, Qingdao 266237, China; 3 Center for Ocean Mega-Science, Chinese Academy of Sciences, Qingdao 266071, China; 4 Yellow Sea and East Sea Buoy Observation Station, Institute of Oceanology, Chinese Academy of Sciences, Qingdao 266071, China; jiasy@qdio.ac.cn; 5 University of Chinese Academy of Sciences, Beijing 100049, China

**Keywords:** microalgae, DNA/RNA stability, resting cysts, sediments, metabarcoding, quantitative real-time PCR

## Abstract

The ever-increasing applications of metabarcoding analyses for environmental samples demand a well-designed assessment of the stability of DNA and RNA contained in cells that are deposited or buried in marine sediments. We thus conducted a qPCR quantification of the DNA and RNA in the vegetative cells of three microalgae entrapped in facsimile marine sediments and found that >90% of DNA and up to 99% of RNA for all microalgal species were degraded within 60 days at 4 °C. A further examination of the potential interference of the relic DNA of the vegetative cells with resting cyst detection in sediments was performed via a metabarcoding analysis in artificial marine sediments spiked with the vegetative cells of two Kareniaceae dinoflagellates and the resting cysts of another three dinoflagellates. The results demonstrated a dramatic decrease in the relative abundances of the two Kareniaceae dinoflagellates in 120 days, while those of the three resting cysts increased dramatically. Together, our results suggest that a positive detection of microalgae via metabarcoding analysis in DNA or RNA extracted from marine sediments strongly indicates the presence of intact or viable cysts or spores due to the rapid decay of relic DNA/RNA. This study provides a solid basis for the data interpretation of metabarcoding surveys, particularly in resting cyst detection.

## 1. Introduction

Genomic analyses of DNA from natural environments present unprecedented opportunities for increasing our understanding of natural microbial biodiversity, processes, and evolution and the functions of different microbial groups [1,2,3]; however, such culture-independent studies of biodiversity and processes usually include extracellular DNA [4] or DNA from fragmental cells since extracellular and fragmental-cell-contained DNA molecules are released and exist in most terrestrial and aquatic environments [4,5]. Dell’Anno and Danovaro [6] provided evidence that most DNA in deep-sea sediments was extracellular. Extracellular DNA is co-extracted with nucleic acids released from lysed cells during sample processing, which may lead to the misinterpretation of the composition of the target living community [5,7,8]. Therefore, ribosomal RNA (rRNA), instead of rDNA, has been frequently used toward the goal of identifying populations that are currently active in a mixed community during the past two decades because RNA abundance generally reflects the metabolic activity of viable cells and RNA is more promptly degradable than DNA [9,10,11,12,13,14,15,16,17,18,19,20,21,22]. However, there is evidence indicating that the general use of rRNA as a reliable indicator of metabolic states in microbial assemblages has limitations as well. Conflicting patterns between the rRNA content and growth rate indicate that rRNA is not a reliable metric for growth or activity and, in some cases, may be grossly misleading [23,24]. This is because propidium monoazide (PMA), as a proposed DNA-intercalating dye, could enter membrane-damaged cells and bind to the DNA after photoactivation, which will prevent the stained DNA from undergoing PCR amplification. Thus, the use of PMA has been recently combined with quantitative PCR (qPCR) to selectively inhibit the PCR amplification of DNA from dead cells and allow for viability-based discrimination [5,25,26,27]. Ramírez et al. [28] used PMA to study the influence of extracellular 16S rRNA on molecular surveys of marine sedimentary communities. However, again, many factors may interfere with the efficiency of PMA used to differentiate live and dead cells, as exemplified by the effects of PMA concentration [29,30] and the ratio of dead to viable cells in the sample [31,32]. Collectively, there seems to be no perfect method to eliminate potential interference from relic DNA/RNA (i.e., extracellular DNA or that in lytic cells) [33] in the environment, although it is known that the integrity of extracellular DNA/RNA molecules exposed to the ambient environment would be severely destroyed by numerous physical and chemical factors [4]. This situation, thus, necessitates a more robust assessment of the stability of DNA and RNA contained in different types of cells in the environment.

The possible interference of relic DNA or fragmental cells has also been a challenging question raised with respect to the detection of resting cysts (spores and resting-stage cells) of microalgae, dinoflagellates in particular, from marine sediment using the metabarcoding approach. Microalgae not only act as some of the most important primary producers but also play vital roles in the bioremediation of anthropogenic pollutants from water [34]. Resting cysts, representing a dormant stage in the life history of microalgae, maintain viability from months to decades or even centuries [35,36,37,38] in sediment and play vital roles in the ecology of dinoflagellates and other microalgae, particularly in initiating and terminating harmful algal blooms (HABs) [35] and in expanding geographic distribution [39,40]. Since only ~200 out of the 2400 accepted species of dinoflagellates were reported to produce resting cysts [41,42,43,44,45], it is therefore highly desirable to establish inventories of resting cyst assemblages of dinoflagellates, particularly those that were not reported as resting cyst producers in coastal countries in general and, in particular, water bodies and regions of importance (e.g., those with intensive aquaculture or that are prone to forming HABs). The identification of cysts in sediment samples was traditionally based on morphological observation under a light microscope (rarely using SEM). However, morphological identification has numerous limitations for species of tiny sizes that were not taxonomically described, lack reference micrographs, or lack distinctive taxonomic characters [45]. Metabarcoding analysis thus provides a powerful tool for determining resting cyst diversity [46,47,48,49,50]. However, these investigations face challenging questions regarding the possible interferences of relic DNA with the data interpretations of metabarcoding. Therefore, the stability of relic DNA and RNA (i.e., how long they can remain) in the natural environment is a critical issue that deserves carefully designed examinations given that only a few experimental investigations were available in the literature that were targeted to assess the effect of relic DNA/RNA on microbial diversity [33,51]. However, experimental investigations into the effect of the relic DNA/RNA of marine microalgae on the identification of resting cysts (spores) in sediments via metabarcoding analyses have been virtually absent.

Thus, in this study, facsimiles of marine sediment (referred to as type I sediments hereafter) were created in the laboratory to which live cells of three microalgal species (*Aureococcus anophagefferens*, strain CCMP1984; *Akashiwo sanguinea*, strain ASND01; and *Scrippsiella acuminata* = *Scrippsiella trochoidea* in Loeblich, 1976 [52] (see Kretschmann et al., 2015 [53]), strain STIOCAS01) were added, and quantitative measurements of relic large subunit (28S) rDNA and rRNA were then conducted via quantitative real-time PCR in a time series of cold storage lasting up to 60 days. Additionally, we conducted an examination of the potential interference of the stability of relic vegetative cell DNA in the detection of resting cysts by entrapping the vegetative cells of two dinoflagellates (*Karenia mikimotoi*, strain KMND02 and *Karlodinium veneficum*, strain KVND01) and the resting cysts of three dinoflagellates (*S. acuminata*, strain STIOCAS01; *Alexandrium andersonii*, strain AADD01; and *Gymnodinium microreticulatum*, strain GMLYG03) in facsimile marine sediments (referred to as as type II sediments hereafter), which were then stored in the cold, sampled at different time points, and measured via metabarcoding for the detection of resting cysts. The choice of 28S rDNA and rRNA was based on the fact that this gene has been increasingly used in the taxonomy, phylogenetic analyses, and identification of eukaryotic microorganisms since 2000 [54,55].

## 2. Results

### 2.1. Stability of DNA and RNA of Three Marine Microalgae in Cold-Stored Type I Sediments

**Figure 1:**
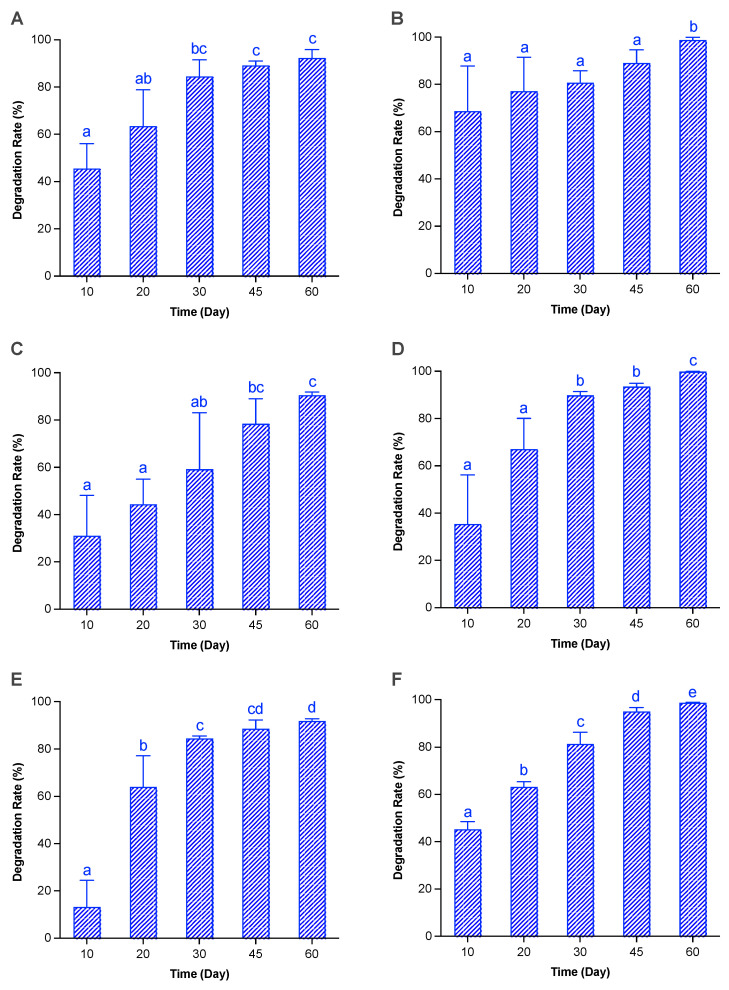
Degradation rates (%) of the targeted microalgal DNA (**A**,**C**,**E**) and RNA (**B**,**D**,**F**) fragments in type I sediment samples along with the storage time (*Au. anophagefferens*: (**A**,**B**); *Ak. sanguinea*: (**C**,**D**); *S. acuminata*: (**E**,**F**)). Type I sediments were the facsimile marine sediments entrapping three microalgae: *Au. anophagefferens*, *Ak. sanguinea*, and *S. acuminata*. The small letters indicate the statistical significance of differences among the treatment groups.

We conducted a quantitative examination of the stability of DNA and RNA via entrapping the vegetative cells of three microalgae (*Au. anophagefferens*, *Ak. sanguinea*, and *S. acuminata*) in facsimile marine sediments (type I sediments) which were stored in the cold (at 4 °C to imitate natural winter conditions and slow down the degradation of DNA and RNA), sampled at different time points, and measured for DNA/RNA content via quantitative real-time PCR. For all the three microalgae, their coefficients of determination (R^2^) for the DNA standard curves were higher than 0.999 (0.9991–0.9998), and the amplification efficiencies varied from 95.52% to 96.96%, indicating a sound linear relationship between the copy number of the targeted gene and the Ct value (Figure S1A,C,E). A longer time of storage in a 4 °C fridge resulted in significantly lower DNA content and thus higher DNA degradation in all three microalgae (Figure 1A,C,E). Within 30 days, the DNA degradation of *Au. anophagefferens* and *S. acuminata* reached more than 80%, and that of *Ak. sanguinea* reached about 60%. In 60 days, the DNA degradation of all three species reached 90–92% (*Au. Anophagefferens*, 92.33%; *Ak. Sanguinea,* 90.50; *S. acuminata*, 92.10%). Regarding rRNA degradation, the coefficients of determination (R^2^) for the cDNA standard curves of all three microalgae were more than 0.999 (0.9995–0.9999), and the amplification efficiency ranged from 94.77% to 99.77%, indicating a sound linear relationship between the copy number of the targeted gene and the Ct value (Figure S1B,D,F). Again, longer storage at 4 °C led to significantly higher rRNA degradation for all three microalgae (Figure 1B,D,F), and the degradation of rRNA reached up to 98–99% in 60 days of storage (*Au. Anophagefferens*, 98.75%; *Ak. Sanguinea,* 99.87%; *S. acuminata*, 98.72%). Faster than that of rDNA, the degradation of rRNA for all three microalgae exceeded 80% in 30 days (Figure 1B,D,F). After 60 days of storage, 8–10% of rDNA and 1–2% of rRNA still existed in the sediments for these microalgae. Based on our previous measurements of the copy numbers of the 28S rRNA gene per cell of *Ak. sanguinea* and *S. acuminata* [56], after 60 days of storage at 4 °C, the cell numbers of *Ak. sanguinea* and *S. acuminata* were calculated to decrease from 5000 cells and 2500 cells to 406 cells and 237 cells, respectively. Note that *Au. anophagefferens* was not included in the study [56].

### 2.2. Interference of Relic Vegetative Cell DNA with the Detection of Resting Cysts via Metabarcoding Analyses

To examine the possible interference of the stability of relic DNA of microalgal vegetative cells in the detection of resting cysts in sediments, we surveyed resting cyst diversity in the type II artificial marine sediments that were made of terrestrial soil and filtered seawater and were then spiked with the vegetative cells of two Kareniaceae dinoflagellates (*Kare. mikimotoi* and *Karl. veneficum*) and the resting cysts of three dinoflagellates (*S. acuminata*, *Al. andersonii*, and *G. microreticulatum*), with the spiked sediments stored at 4 °C and sampled at different time points for metabarcoding analyses.

#### 2.2.1. An Overview of the Metabarcoding Sequences

The Good’s coverage values of all samples virtually approached 1.00, and each of the curves of Good’s coverage and rarefaction reached a plateau (Figure S2), suggesting sufficient sequencing depth for all samples. A total of 1,733,795 raw tags were generated from 21 samples, and 1,611,949 valid tags were obtained after filtering low-quality reads, trimming the adapters and barcodes, and removing chimeras. All these valid tags were clustered into 584 ASVs, and 1,605,096 valid tags (99.57%) were annotated into the target five dinoflagellates.

#### 2.2.2. Composition and Structure of the Communities

**Figure 2:**
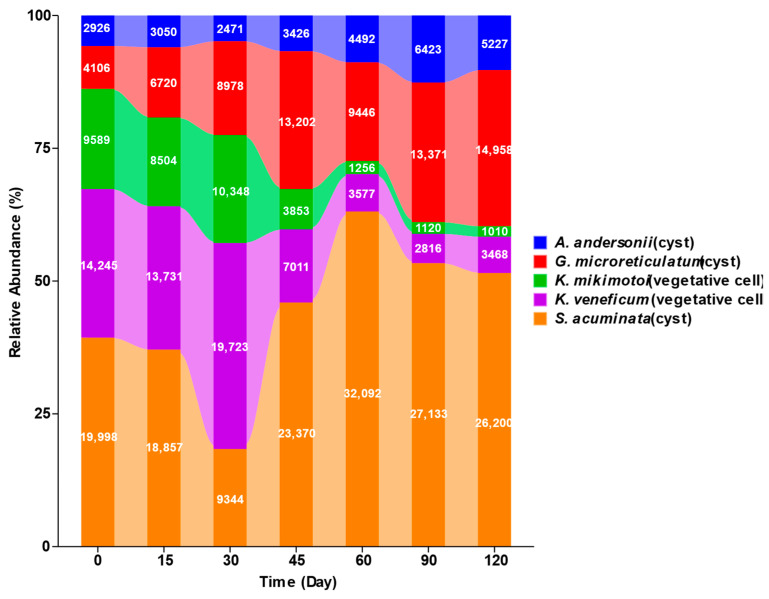
Changes in the relative abundances of the five microalgae spiked in type II sediments along with the storage time. The numbers on the columns are the read numbers of the respective microalgal species. Type II sediments were facsimile marine sediments that were made of terrestrial soil and filtered seawater and then spiked with the vegetative cells of two Kareniaceae dinoflagellates (*Kare. mikimotoi* and *Karl. veneficum*) and the resting cysts of another three dinoflagellates (*S. acuminata*, *Al. andersonii*, and *G. microreticulatum*).

The compositions and relative abundances of the type II sediment samples with different storage periods are shown in Figure 2. The relative abundance of the *Kare. mikimotoi* vegetative cells ranged from 16.71% to 20.34%, and that of the *Karl. veneficum* vegetative cells varied from 27% to 38.75%, together accounting for about 50% (49.88 ± 8.13%) within 15 and 30 days of cold storage, while the summed relative abundances of the three species of resting cysts (*S. acuminata*, *Al. andersonii*, and *G. microreticulatum*) accounted for the other 50%. After 30 days, the relative abundances of both *Kare. mikimotoi* and *Karl. veneficum* displayed dramatic decreases and reached as low as 1.99% and 5.54%, respectively, after 120 days of cold storage, while the summed relative abundances of the three resting cyst species reached up to 92.47% on Day 120, with an average of 88.15 ± 8.13%.

**Figure 3:**
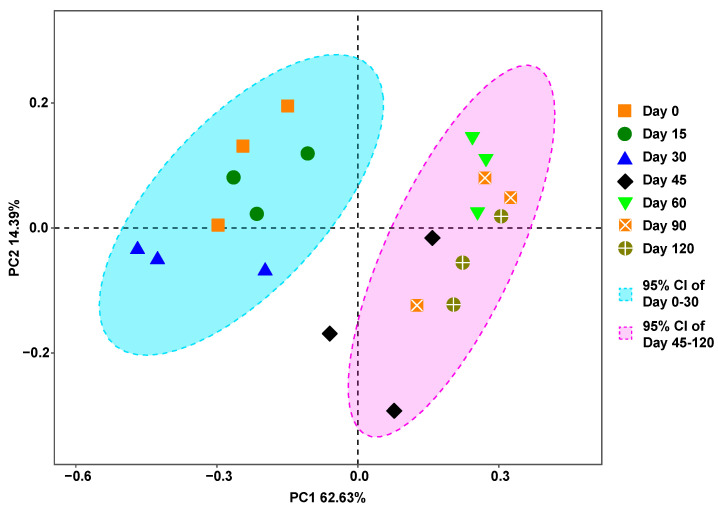
Principal coordinate analysis (PCoA) of all type II sediment samples (CI: confidence interval). Type II sediments were facsimile marine sediments that were first made of terrestrial soil and filtered seawater and then spiked with the vegetative cells of two Kareniaceae dinoflagellates (*Kare. mikimotoi* and *Karl. veneficum*) and the resting cysts of another three dinoflagellates (*S. acuminata*, *Al. andersonii*, and *G. microreticulatum*).

The results of the PCoA showed that all samples formed two clusters: one for the samples cold-stored within 30 days, the other for those cold-stored for 45 to 120 days, although the data points for Day 45 were somehow loosely expanded (Figure 3). The PCoA results indicated that no significant difference in community structure was observed among the samples that were cold-stored for 30 days and less and among those samples that were stored for 45 days and longer, while the samples stored for 0-30 days were significantly different in community structure from those stored for 45 days to 120 days, which corroborates the results in Figure 2 that the relative abundances of both the *Kare. mikimotoi* and *Karl. veneficum* vegetative cells dramatically declined after the storage time exceeded 30 days.

#### 2.2.3. Stability of DNA in the Vegetative Cells of Two Kareniaceae Species in Type II Sediments Measured via qPCR 

**Figure 4:**
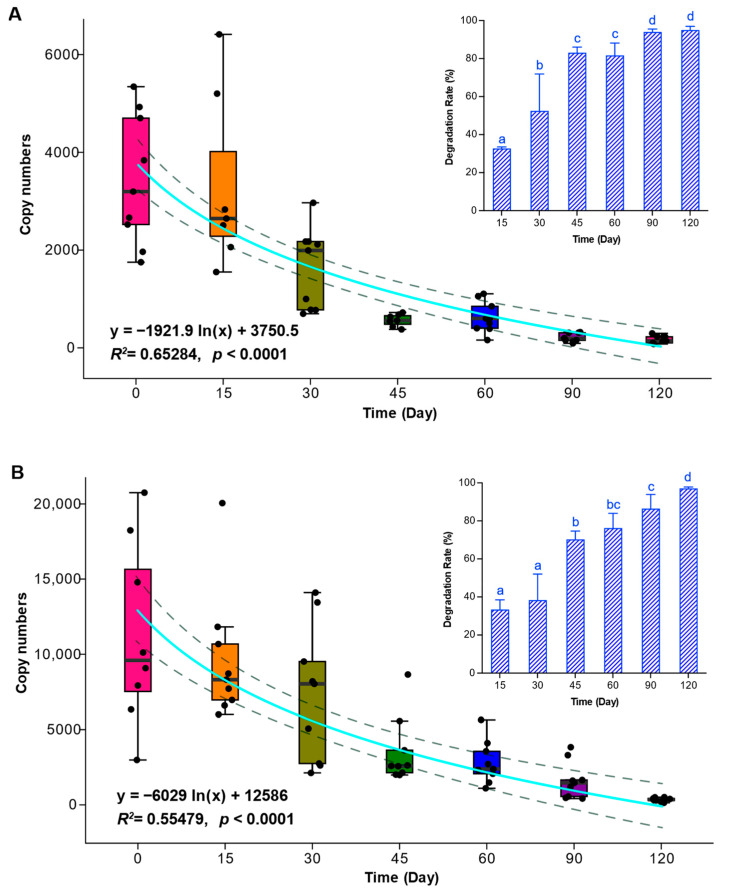
Correlation between copy numbers of 28S rRNA genes of *Kare. mikimotoi* (**A**) and *Karl. veneficum* (**B**) and storage time and the degradation rates of *Kare. mikimotoi* (the insert in (**A**)) and *Karl. veneficum* (the insert in (**B**)) along with the collapse of storage time. The small letters above the columns represent the statistical significance of differences among the treatment groups.

Using procedures similar to those used for the type I sediments, an additional examination of the stability of the rDNA of two Kareniaceae vegetative cells was conducted via quantitative real-time PCR, aiming to verify the results of the metabarcoding analyses. For the two Kareniaceae dinoflagellates, their coefficients of determination (R^2^) for the DNA standard curves were almost 0.999 (0.9979 and 0.9987), and the amplification efficiencies were 96.56% and 97.00%, respectively, indicating a sound linear relationship between the copy number of the targeted gene and the Ct value (Figure S3A,B). The copy numbers of the 28S rRNA gene of both *Kare. mikimotoi* and *Karl. veneficum* showed exponential decreases (R^2^ = 0.65 and 0.55, respectively; *p* < 0.0001 for both species) during 120 days of cold storage (Figure 4), indicating that longer storage in a 4 °C fridge resulted in significantly higher DNA degradation, consistent with the results for the three microalgae in the type I sediments above. Within 30 days, the DNA degradation rates of *Kare. mikimotoi* and *Karl. veneficum* were 52.51% and 38.29%, respectively, while the corresponding degradation rates rapidly reached 83% and 70.22% in 45 days (Figure 4), results which were consistent with the above-described results of the changes in relative abundance and PCoA. At the end of 120 days of cold storage, the rDNA degradation rates of *Kare. mikimotoi* and *Karl. veneficum* reached 96.04% and 97.02%, respectively (Figure 4A,B). Based on our previous measurements of the copy numbers of the 28S rRNA gene per cell in *Kare. mikimotoi* and *Karl. veneficum* [56], after cold storage for 120 days, the cell numbers of *Kare. mikimotoi* and *Karl. veneficum* were calculated to decrease from 2000 cells and 5000 cells to 99 cells and 156 cells, respectively.

## 3. Discussion

### 3.1. The Stability of Relic DNA/RNA in the Vegetative Cells of Microalgae in Sediments

The relic DNA/RNA of three microalgae in facsimile sediments used for a quantitative examination of the stability of DNA/RNA (type I sediments containing vegetative cells only) displayed striking degradation within 60 days of cold storage, i.e., the DNA degradation of all three species reached 90–92%, and the RNA degradation reached 98–99%. While the DNA degradation of *Au. anophagefferens* and *S. acuminata* was mostly completed in a month, that of *Ak. sanguinea* showed a slightly lower rate within the period of 60 days, possibly due to a relatively larger variation among replicates (as reflected in the error bar shown in Figure 1C). The qPCR detections targeting the DNA of *Kare. mikimotoi* and *Karl. veneficum* vegetative cells in the type II sediments containing both vegetative cells and resting cysts that were used for metabarcoding analyses revealed similar trends in that the DNA was largely depleted (95–97%) during 120 days of cold storage. Although the relic DNA of the microalgal vegetative cells in both the type I and type II sediments showed a similar decreasing trend, the degradation rate in type II sediments was lower than that in type I sediments (i.e., 50% on day 30 and 80% on day 60 vs. 80% on day 30 and 90% on day 60, respectively). This difference was most possibly due to differences in the initial amount of microalgal biomass and the degradability of different algal species, together with the different species compositions and numbers of algae-degrading bacteria contained in the 2 μm filtered seawater samples that were prepared for the two batches of the experiment at different times, although other factors could not be completely excluded.

Relic DNA (i.e., extracellular DNA), acting as one class of organic molecules, is available for catabolism by microorganisms harbored in natural marine sediments, contributing substantially to oceanic and sedimentary biogeochemical cycles, acting as an important source of carbon, nitrogen, and phosphorus, and providing an energy source [6,57,58]. Estimates suggest that “bioavailable” DNA, i.e., DNA available for digestion by extracellular nucleases, supplies the microbial communities of coastal and deep-sea sediments with 2–4% of their requirements of carbon, 4–7% of their nitrogen requirements, and, remarkably, 20–47% of their phosphorus requirements [6,58]. Previous analyses of prokaryotic ribosomal RNA genes derived from extracellular and intracellular DNA pools in marine sediments showed that extracellular DNA is rapidly turned over in both shallow and deep sediments (down to 10–12 m) [28,59]. All results of previous research studies collectively provide multiple lines of evidence proving that there exists a bulk of DNA-foraging microorganisms in natural marine sediments. Wasmund et al. [60] identified diverse bacterial taxa (e. g., “*Candidatus* Izemoplasma”, *Lutibacter*, *Shewanella*, and Fusibacteraceae) that showed a high capacity for degrading extracellular DNA in marine sediments, as seen in the case in which ^13^C-DNA added to sediment microcosms could be largely degraded within 10 days and mineralized to ^13^CO_2_. Diverse “*Candidatus* Izemoplasma” and Bacilli families encode multiple predicted extracellular nucleases and catabolic enzymes for DNA subcomponents, and extracellular nuclease genes are prevalent among Bacteroidota, conferring them a strong ability to rapidly degrade extracellular DNA in marine sediments [60]. The extracellular DNA of the microalgae in those marine sediments sampled from the field might be degraded much faster than that in our experiments because of the high level of activity of the nucleases in the natural sediments, with temperatures generally higher than that applied in the present study. While the extracellular DNA of microalgae in field marine sediments is controlled both by input (from the dead vegetative cells and/or DNA of microalgae sinking from the photic zone) and degradation (by the bulk of DNA-foraging microorganisms), the extracellular DNA of the microalgae (i.e., the vegetative cells presumably erupted within a few days) in the sediments that were sampled and stored in cold conditions in the present work was solely controlled by the DNA degradation rate. Accordingly, with the degradation trends shown in Figure 1 and Figure 4, the extracellular DNA of the microalgae in the marine sediments sampled from the field would be exhausted if the samples were stored for a longer time, or/and at a higher temperature, or/and under more aerobic conditions.

### 3.2. Assessing Possible Interference of relic DNA of Microalgal Vegetative Cells in Metabarcoding Surveys of Resting Cyst Communities in Marine Sediments

Analyses of the relative abundances and species compositions of the type II sediment samples provided insights into concern about the possible interference of relic DNA from or within the fragmental vegetative cells of microalgae in metabarcoding surveys of resting cyst communities. In the 30 days of cold storage, the sequences of the two Kareniaceae species accounted for almost 50%; however, with an increase in storage time, the relative abundances of the *Kare. mikimotoi* and *Karl. veneficum* sequences showed dramatic decreases and reached as low as 1.99% and 5.54% after 120 days, respectively. The PCoA results showed that the species compositions of the samples stored for 30 days were significantly different from those of the samples stored for longer periods of time. The read numbers of the microalgae on Day 30 and Day 60 are abnormal in Figure 2; regarding the abnormal values on Day 30 and to a lesser extent on Day 60, a most parsimonious explanation is that they were caused by an uneven mixing of different algal species (*Karl. veneficum* in particular) for the tubes that were removed for the subsequent DNA extraction (i.e., a higher initial cell number of *Karl. veneficum* was added to the sediment tube that was sampled on Day 30 and a there was a lower number of *G. microreticulatum* cysts on Day 60), given that a minor difference in cell density could be magnified in the PCR amplification prior to metabarcoding sequencing. However, this irregularity in the temporal trend of *Karl. veneficum* (and, to a lesser extent, *G. microreticulatum*) would not alter the overarching trends for the changes in the relative abundances of the five species, as the samples represented at those different time points were prepared and sampled in separate tubes and thus would not interfere with one another. The reasons mentioned above are responsible for the greater scattering of the Day 45 plots shown in Figure 3 as well. All these results indicate that with an increase in storage time and the degradation of relic DNA in vegetative cells (i.e., extracellular DNA), the interference of relic DNA in microalgal vegetative cells in the metabarcoding surveys of resting cyst communities reduces with time and reaches a negligible level. Sequencing depth is one of the most critical factors determining the reliability of eDNA metabarcoding; a minimum sequencing depth is certainly needed to correctly characterize the diversity of environmental samples [61]. Alberdi et al. [62] compared read depths of ~2500 to ~25,000 reads per replicate and found that alpha diversity increased with sequencing depth. Shirazi et al. [63] found that increasing the read depth from 1000 to 10,000 reads resulted in 1.8-fold and 2.4-fold increases in the apparent alpha diversity for the ITS2 region targeting plants and the ITS1 region targeting fungi, respectively. These results and those of other previous studies allow us to conclude that the higher the sequencing depth, the more taxa will be detected, especially for those with low abundance and low biomass and those strongly affected by primer bias [61,62,63,64]. The sequencing depth of our study with five species was about 50,000 reads, which is equal to or higher than that of other studies targeting natural sediment samples with much higher richness values and abundances of intact or live cells (resting cysts) [46,47,48,49,50], which means that the positive detection of the relic DNA of microalgal vegetative cells via metabarcoding in our study largely resulted from the high sequencing depth, and the relic DNA of microalgal vegetative cells, especially those after 45 days of storage, would be more likely to be undetectable if a lower sequencing depth was applied. As we discussed above, the relic DNA of microalgal vegetative cells in natural sediments will degrade much faster than that in the facsimile sediments in our study due to higher temperatures, oxygen, and degrading microorganisms in the field. Therefore, in other words, the relic DNA of microalgal vegetative cells in natural sediments subjected to storage conditions similar to those in our study will be more likely to go undetected with the same or a lower sequencing depth, that is, the potential interference of the relic DNA of microalgal vegetative cells in natural sediments is minimal or negligible after several months of cold storage, given the commonly adopted sequencing depths. A previous study concluded that the overall effect of extracellular genes isolated by the photoactive DNA-binding dye propidium monoazide on the metabarcoding surveys of marine sedimentary prokaryotes was minimal as well [28]. The identification of cysts in environmental sediment samples has mainly relied on LM and published reference micrographs in which a mesh sieve of 20 μm was usually used [65]. However, many species are very small as both vegetative cells and cysts [38,66], and their cysts could be easily lost during filtration processing. Furthermore, while some different species may be of very similar morphologies, other species of cysts may vary significantly in morphology for the same species [48,67], both of which could lead to misidentification or identification difficulty. For example, *Gymnodinium nolleri* and *G. catenatum* were misidentified due to the morphological similarity of their cysts and vegetative cells [68], while the cysts of both *Alexandrium ostenfeldii* and *Diplopsalis lenticula* from marine sediments varied to a great extent [48]. Therefore, identification based on morphology only has numerous limitations for species of tiny sizes which have not been taxonomically described, lack reference micrographs, or lack distinct diagnostic features [46]. With advancements in molecular techniques in recent decades, metabarcoding analyses have been increasingly applied to the identification and quantification of dinoflagellates [46,47,48,49,50,69,70,71]. While the metabarcoding approach is advantageous in detecting large numbers of samples efficiently and in detecting the assemblages of all known and unknown species present in the samples, a possible drawback is that when the method is applied to cyst detection from sediment samples, a crucial question may be raised regarding whether the retrieved DNA sequences are factually from cysts or otherwise from vegetative cells and/or cell fragments [48]. In our study, the rDNA of the two Kareniaceae species in sediments showed exponential decay with storage time and degraded by more than 95% after 120 days of storage, while the relative abundances of the resting cysts of the three dinoflagellates increased from 39.32%, 5.75%, and 8.07% to 51.51%, 10.28%, and 29.41%, respectively, during the same period of storage. Our results thus suggest that a positive detection of microalgae via PCR-based metabarcoding from the DNA or RNA extracted from sediment samples stored at 4 °C for more than several months or from long-buried field sediments strongly indicates the presence of intact or viable cells (cysts or spores) since the DNA/RNA from vegetative or fragmental cells would have largely decayed within a short time period and their minimal residues would most likely be overwhelmed by those of viable cells due to their higher copies of rDNA or other markers. We believe this study provides a solid basis for a possible strategy to eliminate the interference of relic DNA during experimental design and sample pretreatments for metabarcoding surveys on the diversity and structure of environmental microbial communities.

Theoretically, a trace amount of DNA/RNA (particularly DNA) will still be present in a sample even if the sample was stored for a longer time, and consequently, species-specific primers for PCR amplification will still be able to detect individual species from the trace DNA. However, when more universal primers are used (e.g., massively parallel sequencing (MPS) technologies [3]), only those species with a high abundance or those maintained as viable or intact cells will be detectable simply because those species or cells have more copies or more intact molecules and thus high probabilities of being amplified. In other words, the organisms detected with a PCR using group-specific or universal primers and non-exhaustive pyrosequencing from field samples, particularly those which have been in conditions not suitable for maintaining the intactness of cells or DNA/RNA, will be more likely to be detected in the forms of intact and viable cells such as resting cysts (spores) or cells at a resting stage. 

## 4. Materials and Methods

### 4.1. Microalgal Cultures

Seven clonal microalgae with different sizes, cellular features (e.g., armored or naked), and taxonomic origins were selected from a stock of cultures in the laboratory and used in this study: the brown tide-forming pelagophyte *Aureococcus anophagefferens* (strain CCMP1984 from NCMA) and the red tide-forming dinoflagellates *Akashiwo sanguinea* (strain ASND01), *Scrippsiella acuminata* (*Scrippsiella trochoidea* in Loeblich, 1976 [52] (see Kretschmann et al., 2015 [53]), strain STIOCAS01), *Karenia mikimotoi* (strain KMND02), *Karlodinium veneficum* (strain KVND01), *Alexandrium andersonii* (strain AADD01), and *Gymnodinium microreticulatum* (strain GMLYG03) (established by Y.Z. Tang, Z.X. Hu, and X.Y. Zhai). All the species were identified using partial LSU rDNA sequencing in our previously published paper [72]. The seven microalgae are from two phyla, two classes, four orders, and five families (and seven genera), as follows:

Clade Stramenopiles

 Phylum Gyrista, subphylum Ochrophytinaclass

  Class Dictyochophyceae

   Order Pelagomonadales

    Family, Pelagomonadaceae

     *Aureococcus anophagefferens*

Clade Alveolata

 Phylum Myzozoa, superclass Dinoflagellata

  Class Dinophyceae

   Order Gonyaulacales

    Family Ostreopsidaceae

     *Alexandrium andersnii*

   Order Gymnodiniales

    Family Gymnodiniaceae

     *Akashiwo sanguinea*

     *Gymnodinium microreticulatum*

    Family Kareniaceae

     *Karenia mikimotoi*

     *Karlodinium veneficum*

   Order Peridiniales

    Family Peridiniaceae

     *Scrippsiella acuminata*

The procedures of culture maintenance and the collection of resting cysts of *Al. andersonii*, *G. microreticulatum*, and *S. acuminata* followed Chai et al. [73] and Yue et al. [74] before cells were harvested for experiments, respectively.

### 4.2. Preparation of Artificial (Facsimile) Sediments

Two types of artificial sediments (type I and type II) were designed in this study (Table 1) which encompassed sterilized soil (collected near the IOCAS, Qingdao, to make sure no other marine algae were present in the samples), seawater filtered with a 2 μm filter (to inoculate degrading marine bacteria, exclude other microalgae, and adjust salinity), and mixed target microalgal vegetative cells or resting cysts with proportions of 1 g to 0.01 mL and 0.4 mL, respectively. The artificial sediments were blended using a vortex instrument (sci-vx, SCILOGEX, Knoxville, TN, USA) at 1000 rpm for 1 min. The ratio of the three mixed microalgal cultures (*Au. anophagefferens*, *Ak. Sanguinea*, and *S. acuminata*) of the type I facsimile marine sediment used for the quantitative examination of the stability of DNA/RNA via quantitative real-time PCR was 1:1:1 (the biovolume ratio of microalgal cultures added into the sediments), with the final cell densities of 25 × 10^3^ cells g^−1^, 5 × 10^3^ cells g^−1^, and 10 × 10^3^ cells g^−1^, respectively. The final cell density of the resting cysts in the type II facsimile marine sediments was set to be 50 cysts g^−1^ of each species (*Al. andersonii*, *G. microreticulatum*, and *S. acuminata*), and the vegetative cell densities of the two Kareniaceae dinoflagellates (*Kare. mikimotoi* and *Karl. veneficum*) were 4 × 10^3^ cells g^−1^ and 10 × 10^3^ cells g^−1^, respectively. Amounts of 0.5 g and 2 g each of the artificial sediments were added to pre-sterilized 1.5 mL and 5 mL centrifuge tubes for DNA extraction and RNA extraction separately. All the tubes were sealed to maintain an anaerobic environment during the storage. The tubes were labeled with A1, A2, A3, B1, B2, B3, and so on and were placed in a 4 °C fridge to imitate natural winter conditions and to make the results more conservative (in the sense of slowing the degradation of DNA and RNA). DNA and RNA extractions of the type I sediment samples were conducted on Day 3, Day 10, Day 20, Day 30, Day 45, and Day 60, and DNA extractions of the type II sediments samples were conducted on Day 0, Day 15, Day 30, Day 45, Day 60, Day 90, and Day 120. All samples were obtained in triplicate, and 57 samples were collected in this study. Given that the microalgal vegetative cells could not survive the sediment environments applied in this study, the vegetative cells would be disrupted and subsequently lysed after blending via vortexing after the addition of algal cells to the sediments. The relic DNA referred to in this study, therefore, was the DNA released from the erupted and lytic vegetative cells of the microalgae in the sediments. Considering that the DNA/RNA extracted before mixing with the sediment would be degraded more easily than that initially contained in the vegetative cells, the absence of an experiment using “naked DNA/RNA” should not negatively affect our experimentation.

### 4.3. DNA/RNA Extraction, Primer Design, and Quantitative Real-Time PCR

Total DNA and total RNA were extracted using a FastDNA^®^ SPIN Kit for Soil (MP Biomedicals, Santa Ana, CA, USA) and a PowerSoil^®^ Total RNA Kit (QIAGEN, Dusseldorf, Germany), respectively. The quantity and quality of the total DNA/RNA was measured with a ND-2000 Nanodrop spectrophotometer (Thermo Fisher Scientific, Waltham, MA, USA). First-strand cDNA was prepared with random primers using a SuperScript^®^ III First-Strand Synthesis System (Thermo Fisher Scientific, Waltham, MA, USA) according to the manufacturer’s instructions and then stored at −20 °C. The specific primers of the three species microalgae were designed from the D1-D2 domain of the LSU rDNA (Table 2). The qPCR reactions for the DNA/RNA extracted from the type I sediments were performed using an SYBR^®^ Premix Ex TaqTM (TaKaRa, Tokyo, Japan) on a Bio-Rad CFX96 real-time PCR detection system (Bio-Rad Laboratories, Hercules, CA, USA) with the following program: preheating at 94 °C for 5 min, 39 cycles of denaturing at 94 °C, annealing at 45 °C, 50 °C, or 55 °C (Table 2) for 30 s, and extending at 72 °C for 30 s. Each reaction was performed in a 20 μL volume containing 2 μL of a template (DNA or cDNA), 0.2 μL of each primer (10 μM), 10 μL of 2 × SYBR^®^ premix Ex TaqTM II (TaKaRa, Tokyo, Japan), and 7.6 μL of RNase-free water. The LSU rDNA copy numbers of two Kareniaceae vegetable cells in type II sediments were quantified via Taqman-qPCR assays. The set of specific primers and probes were the same as in our previous studies [55,56] and are shown in Table 2. Real-time PCR experiments were performed using a Light Cycler 480 II real-time PCR Detection System with the following program: preheating at 94 °C for 5 min, 40 cycles of denaturing at 94 °C, annealing at 60 °C for 30 s, and extending at 72 °C for 30 s. Each reaction was performed in a 20 μL volume containing 2 μL of a template, 0.8 μL of each primer (10 μM), 0.8 μL probe (10 μM), 10 μL of 2 × premix Ex Taq II (Japan), and 5.6 μL of RNase-free water. The specificity of each pair of primers was confirmed by dissociation curves. Biological triplicate reactions together with negative controls of each gene were performed on a single plate. The targeted DNA/cDNA gene fragments of each microalgal species (i.e., those in lytic vegetative cells and those in the supposedly live cysts) were cloned according to Chen et al. [57]. Plasmid DNA was extracted with a Plasmid Kit (TaKaRa, Tokyo, Japan) and measured with an ND-2000 Nanodrop spectrophotometer (Thermo Fisher Scientific, Waltham, MA, USA); it was then converted into copies using the formula C = 6.02 × 10^23^ × (concentration of recombinant plasmid (ng∙μL^−1^) × 10^−9^/length of recombinant plasmid (bp) × 660). Ten-fold serial dilutions of a known copy number of the plasmid of the targeted gene clone from the sediments were generated to produce a standard curve over six orders of magnitude per assay. The parameter Ct (threshold cycle) was determined as the cycle number at which a statistically significant increase in reporter fluorescence was detected. qPCR amplification efficiency was determined using the slope of a linear regression model [58] and calculated according to the following equation: amplification efficiency = (10^[−1/slope]^ − 1) × 100 [59].

### 4.4. Metabarcoding Sequencing

#### 4.4.1. Primer Design, DNA Extraction, and Sequencing

The primer pair (DinoF primer, 5′-KACTTTGRRAAGAGAGTTAAAW-3′; DinoR, 5′-TCYGTGTTTCAAGACGGGTC-3′), which was the same as in our previous study [48], was designed against the eukaryotic partial 28SrDNA D1-D2 region. Total genomic DNA was extracted from each type II sediment sample using the Fast DNA SPIN Kit for Soil (MP Biomedicals, Santa Ana, CA, USA), according to the manufacturer’s protocol. The concentration and quality of the extracted DNA were measured using an ND-2000 NanoDrop spectrophotometer (Thermo Fisher Scientific, Somerset, NJ, USA). Barcoded 28S rRNA gene amplicons were sequenced on the NovaSeq-PE250 platform by LC-Bio Technology Co., Ltd. (Hangzhou, China).

#### 4.4.2. Sequencing and Bioinformatics Analyses

Sequencing results were deposited at the National Center for Biotechnology Information (NCBI) under the project number PRJNA1000282. The clean data were obtained after data denoising and the removal of chimeras via DADA2 in QIIME-II with default parameters [75,76]. The sequencing results of all samples were randomly subsampled to the lowest number of reads. The taxonomical assignment of amplified sequence variants (ASVs) was carried out using BLAST against the NCBI NT database. A principal coordinate analysis (PCoA) based on the Jaccard distance was performed with vegan package in R (version 4.3.2) [77]. The relative abundance and PCoA results were visualized using ggplot2 package in R (version 4.3.2) [78].

### 4.5. Statistical Analyses

The degradation rate (%) was calculated according to this equation: degradation rate (%) = (initial copy numbers of targeted DNA/RNA fragment—copy numbers of targeted DNA/RNA fragment at one timing)/initial copy numbers of targeted DNA/RNA fragment × 100. “Degradation” may be understood as the processes that caused the portion of “non-PCR-amplifiable DNA/RNA in the initial DNA/RNA” in the present study. The significance of the variance in the degradation rates of the targeted microalgal DNA/RNA fragments was tested using a one-way ANOVA or *t*-test, using the software SPSS 22.0. The significance level was set at 0.05 for all tests unless otherwise stated.

## 5. Conclusions

In summary, our detections from facsimile marine sediments displayed that the copy numbers of the 28S rRNA gene of microalgal vegetative cells showed a significantly exponential decrease with elapsed time, and both the rDNA and rRNA in the dead vegetative cells of microalgae would be degraded by more than 95% within four months and 98% within two months if kept at 4 °C, respectively. These results suggest more degradation during a longer storage period and faster degradation under conditions with higher temperatures and/or abundant bacteria and/or more oxygen. And the metabarcoding results indicated that with an increase in storage time, the interference of the relic DNA in microalgal vegetative cells in the metabarcoding surveys of resting cyst assemblages in natural sediment samples will be minimal with a longer storage period. Our results together provide multiple lines of evidence indicating that positive detections with a PCR using group-specific or universal primers and metabarcoding sequencing from field samples that have been stored for months to years will be more likely occur the forms of intact and viable cells such as resting cysts (spores). We believe this work provides a solid basis for future investigations involving the assessment of DNA/RNA stability and a possible strategy to eliminate the interference of relic DNA during experimental design and sample pretreatments for metabarcoding analyses of the diversity and structure of microbial communities.

## Figures and Tables

**Table 1:** Microalgae compositions and purposes of two types of facsimile marine sediments in the present study.

Sediments Type	Vegetative Cells and Cell Densities (Cells g^−1^)	Cysts and Cell Densities (Cells g^−1^)	Purposes
Type I	*Aureococcus anophagefferen*, 25 × 10^3^ *Akashiwo sanguinea*, 5 × 10^3^ *Scrippsiella acuminata*, 10 × 10^3^	None	Used for a quantitative examination of the stability of DNA/RNA in relic microalgal vegetative cells
Type II	*Karenia mikimotoi*, 4 × 10^3^ *Karlodinium veneficum*, 10 × 10^3^	*Alexandrium andersonii*, 50 *Gymnodinium microreticulatum*, 50 *Scrippsiella acuminata*, 50	Used for an examination of the potential interference of the stability of relic DNA in microalgal vegetative cells in the detection of resting cysts in sediments via metabarcoding analyses

**Table 2:** Information for the qPCR primers and probes of 5 microalgae used in the present study.

Target Gene (LSU)		Primers (5′ to 3′) and Taqman-Probes	Amplicon Length (bp)	Annealing Temperature
*Au. anophagefferens*	DNA cDNA	F: CCGAACGGCAGAAGTGGTGA R: GACCTTCCCATGAACGACTCCC	116	50 °C (DNA) 55 °C (cDNA)
*Ak. sanguinea*	DNA cDNA	F: CACCAGCAACCGATCTGTCAACT R: CCCCTGGCTTTGCCCTACAC	101	50 °C (DNA) 50 °C (cDNA)
*S. acuminata*	DNA	F: TGTAGTAAGTCTTGAGCAGGACA R: ATCCACAAATGAGTTCCAGCAAACACA	139	45 °C
	cDNA	F: TGATTGCTTGCTGCTTCAAC R: ATCCACAAATGAGTTCCAGCAAACACA	167	50 °C
*Kare. mikimotoi*	DNA	F: GCTTCTCGCCTTGCATGTCAACGTC P: GTTCAACTGAGGACATTCAGTCACT R: TGGCACCAACAACCTTCATGCAGAG	186	60 °C
*Karl. veneficum*	DNA	F: CTTCTTGGTGAGATTGTTGTGCGC P: TGCGCGTCATGCTCAAAACC R: GCGAGYAATTAACCATGTCCCTAGA	158	60 °C

F: forward primer; R: reverse primer; P: probe.

## Data Availability

The raw sequence data have been deposited in the National Center for Biotechnology Information database (https://www.ncbi.nlm.nih.gov/ (accessed 12 December 2023)) (accession No. PRJNA1000282).

## Supplementary Materials

The following supporting information can be downloaded at https://www.mdpi.com/article/10.3390/ijms25031724/s1.
