# Supplementary material for: DNA and RNA Stability of Marine Microalgae in Cold-Stored Sediments and Its Implications in Metabarcoding Analyses
Source: Int J Mol Sci. 2024 Jan 31;25(3):1724. doi: 10.3390/ijms25031724 (PMC10855355; doi:10.3390/ijms25031724)
Supplement: Supplementary file 1 [file ijms-25-01724-s001.zip › ijms-2801482-supplementary.pdf]

Supplemental Information for:

DNA and RNA stability of marine microalgae in cold-stored sediments and its implications in metabarcoding analyses

Zhaoyang Chai, Yuyang Liu, Siyang Jia, Fengting Li, Zhangxi Hu, Yunyan Deng, Caixia Yue, Ying Zhong Tang*

Table of Contents

**Supplementary Figure S1**	Page 2
**Supplementary Figure S2**	Page 3
**Supplementary Figure S3**	Page 3

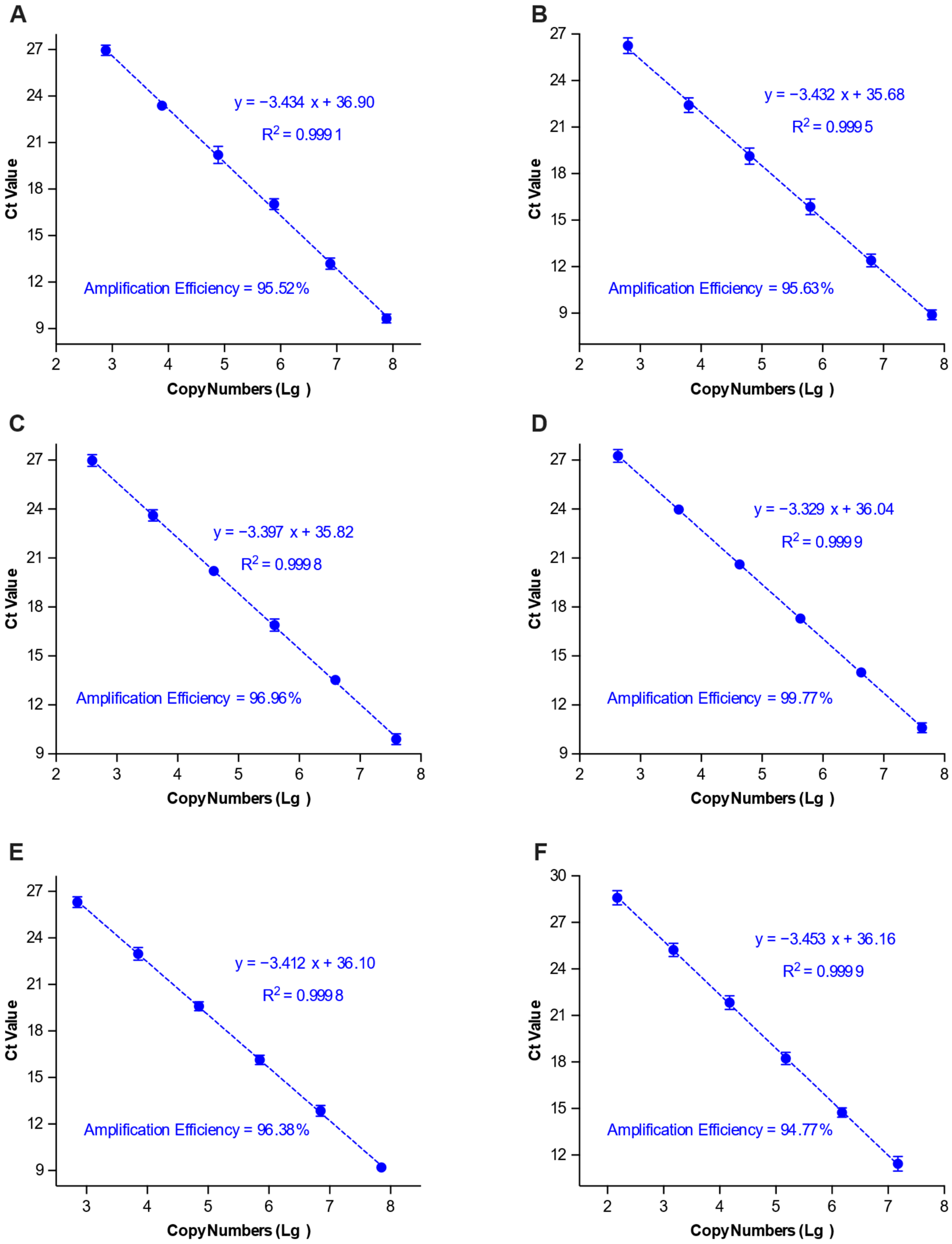

Figure S1. Standard curves for copy numbers of the rDNA (A, C and E) and rRNA (B, D and F) fragment of targeted microalgae in type I sediments (*Aureococcus anophagefferens*: A and B; *Akashiwo sanguinea*: C and D; *Scrippsiella acuminata*: E and F) (n = 3).

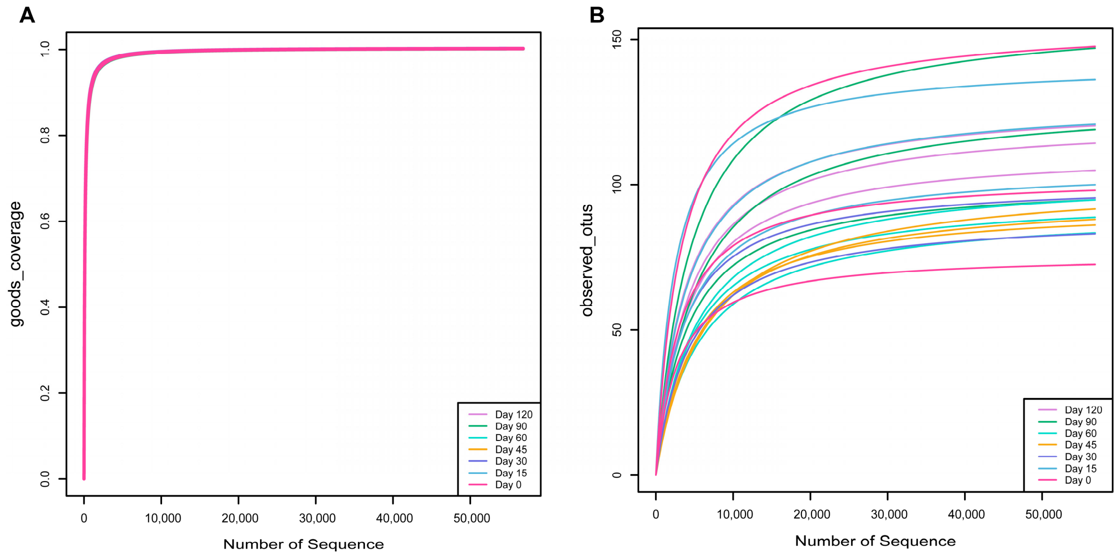

Figure S2. Curves of goods coverage (A) and rarefaction (B) for the metabarcoding sequencing of the type II sediment samples.

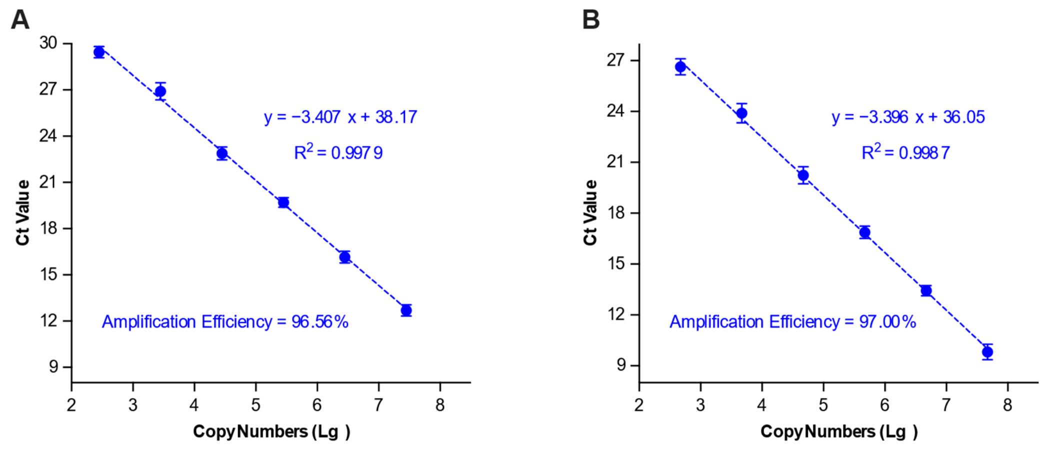

Figure S3. Standard curves for copy numbers of the rDNA fragment of *Karenia mikimotoi* (A) and *Karlodinium veneficum* (B) conducted for the type II sediment samples (n = 3).
